# Nanotribology

**DOI:** 10.3762/bjnano.9.217

**Published:** 2018-08-28

**Authors:** Enrico Gnecco, Susan Perkin, Andrea Vanossi, Ernst Meyer

**Affiliations:** 1 Otto Schott Institute of Materials Research (OSIM), Friedrich Schiller University Jena, Löbdergraben 32, 07743 Jena, Germany; 2 Department of Chemistry, Physical and Theoretical Chemistry Laboratory, University of Oxford, Oxford OX1 3QZ, United Kingdom; 3 CNR-IOM Democritos National Simulation Center, Via Bonomea 265, 34136 Trieste, Italy; 4 International School for Advanced Studies (SISSA), Via Bonomea 265, 34136 Trieste, Italy,; 5 Department of Physics, University of Basel, Klingelbergstr. 82, CH-4056 Basel, Switzerland

**Keywords:** nanotribology, nanoadhesion, nanofriction

Nanotribology is a young and dynamic field of research which aims to investigate friction, wear and adhesion phenomena down to the nanometer scale. Since these phenomena occur in all natural, artificial or conceptual situations involving two surfaces (at least) in contact or in close proximity to each other, it is not surprising that, knowingly or not, many physicists, materials scientists, mechanical engineers or chemists have to contend with these topics sooner or later in their careers.

This Thematic Series is intended as an “invitation to nanotribology” addressed to the attentive readership of the *Beilstein Journal of Nanotechnology*. The first goal of this Thematic Series is simply to make more colleagues and students aware of the existence of this fascinating subject, which, in spite of its universality, is often considered as a niche topic even at prestigious scientific conferences. The second goal, which is closely related to the first one, is to provide fresh examples of the most recent advancements in the field worldwide. This is substantiated in a series of ten original contributions whose authors are quite well distributed around the world (from India, China, Argentina, Cameroon, Russia and USA back to many countries in Old Europe).

The covered topics include lubrication, surface preparation and theoretical models of friction at the nanoscale. Regarding the first topic, this Thematic Series gives examples of cutting-edge aqueous solutions including nanodiamonds [1] and novel materials such as nitrogen-doped graphene oxide [2] and imidazolium-based ionic liquids [3] used as additives to mineral oils. Standard large-scale applications to steel surfaces, but also to a material of key importance in micro- and nanoelecromechanical systems, i.e., silicon oxide, are recognized. The quality of the surface condition is addressed experimentally by the example of cryogenically treated martensitic stainless steel [4] and theoretically by an analysis of the influence of micro-dimple textures on hydrodynamic lubrication [5]. On a more fundamental level, different authors have modeled the influence of electrical double layers on hydrodynamic lubrication [6], the occurrence of a second-order phase transition in ultrathin lubricant films [7] and the velocity dependence of dry friction on crystal surfaces at the atomic scale [8].

While many experimental techniques for materials characterization are those typical of surface science (e.g., X-ray diffraction, SEM, TEM, XPS and Raman spectroscopy), more specific to nanotribology are nanoindenters, nanotribometers, quartz force microbalance and especially atomic force microscopy (AFM), which, without a doubt, has triggered a true revolution in our understanding of friction at the atomic scale. This is exemplified by the lattice resolved friction force images on oxidized silicon surfaces in the tribochemistry study presented by the Bennewitz group [9]. On a larger scale, alternative surface scan methods (e.g., circular mode AFM) for investigation of abrasive wear are proposed by Noel et al. [10].

Considering the interdisciplinary nature of the subject, and the variety of materials, lubricants, and possible applications, the previous examples, in spite of their high quality, are still not enough to achieve our goals. For this reason, the research articles in this Thematic Series are complemented by a rather comprehensive but concise review paper [11], which was born out of the collaboration of nine researchers (experimentalists and theoreticians) within the European COST Action MP 1303 “Understanding and Controlling Nano and Mesoscale Friction”, which ran from 2013 to 2017. Here the covered topics include but are not limited to controlled manipulation of nanoparticles, optically trapped colloidal and ionic systems, superlubricity of graphene, sliding friction of organic molecules, charge density waves, and perspectives of tuning friction using photo-assisted reactions.

Hopefully, new ideas and further research work will be stimulated from this Thematic Series, which could not have come to light without the contributions of all authors and the constant support from the Beilstein-Institut and specifically, Dr. Wendy Patterson and Dr. Uli Fechner.

Enrico Gnecco, Susan Perkin, Andrea Vanossi, and Ernst Meyer

Jena, Oxford, Trieste and Basel, August 2018
